# Economic evaluation of ceftazidime-avibactam vs. polymyxin B for treatment of hospital-acquired and ventilator-associated bacterial pneumonia

**DOI:** 10.1016/j.bjid.2025.104545

**Published:** 2025-05-14

**Authors:** Jessica Matuoka, Daniela Vianna Pachito, Filipe Piastrelli, Lorena Cristina Correa Fehlberg, Haliton Alves de Oliveira Junior

**Affiliations:** a Hospital Alemão Oswaldo Cruz, São Paulo, SP, Brazil; b Pfizer Brasil, São Paulo, SP, Brazil

**Keywords:** Pneumonia, Ventilator-associated, Ceftazidime-avibactam, Polymyxin B, Cost-effectiveness analysis, Critical care, Carbapenem-resistant

## Abstract

Ventilator-associated pneumonia is one of the most common infections in Intensive Care Units (ICU). It is frequently caused by multidrug-resistant pathogens (including carbapenems) and is an important health issue. It may result in severe clinical consequences, with higher healthcare utilization and high economic burden. Timely and appropriate treatment is key to obtaining better outcomes and allocational efficiency. Currently, the treatment options for carbapenem-resistant pathogen infections are limited, usually based on polymyxin, aminoglycosides, or combination therapy, as well as novel antibiotic therapies including Ceftazidime/Avibactam (CAZ-AVI). CAZ-AVI has shown activity against gram-negative pathogens and is currently used for the treatment of Ventilator-Associated Pneumonia (VAP). To better inform healthcare professionals and help promote a rational use of antibiotic therapy, a cost-effectiveness analysis was conducted to compare the cost-effectiveness of CAZ-AVI versus polymyxin B in ICU patients with VAP from the Brazilian National Supplementary Health Agency perspective over a 5-year time horizon. CAZ-AVI had higher total costs and resulted in more Quality-Adjusted Life Years (QALY) gained when compared with polymyxin B. At a willingness-to-pay threshold of BRL 40,000.00/QALY gained, CAZ-AVI was the cost-effective strategy (ICER: BRL 35,298.65/QALY gained). Nephrotoxicity in patients treated with polymyxin B, hospitalization utility, and treatment duration were the variables that most influenced the results. In the probabilistic sensitivity analysis, CAZ-AVI was cost-effective in 55 %–89 % of the interactions. The evidence suggests that CAZ-AVI results in lower mortality and nephrotoxicity rates, which might have contributed to more QALYs gained and a favorable ICER, despite the higher costs. This study was registered on the Open Science Framework database (Protocol https://doi.org/10.17605/OSF.IO/SP2EJ).

## Introduction

Ventilator-Associated Pneumonia (VAP) describes pneumonia that develops in patients exposed to mechanical ventilation for at least 48 hours.^1^ It is one of the most frequent infections in Intensive Care Units (ICUs) in Europe, in the United States,^1^ and in Brazil.^2^ VAP, by itself, is associated with an increased risk of mortality and morbidity and imposes higher healthcare utilization and high economic burden.^1^^,^^3^^,^^4^ When caused by Multidrug-Resistant Pathogens (MDR), worse outcomes can be expected.^3^

Treatment delays or inappropriate prescription of antibiotics contribute to increased mortality risk and costs of treatment.^4^ Therefore, empiric therapy is prescribed before determining the pathogen.^5^

However, with the unreasonable use of carbapenems, antibiotic resistance has been increasing. Currently, the treatment options for carbapenem-resistant pathogen infections are limited, usually based on polymyxin, aminoglycosides, or combination therapy, as well as novel antibiotic therapies including Ceftazidime/Avibactam (CAZ-AVI).^6^ However, polymyxin B and aminoglycoside drugs have been associated with nephrotoxic side effects.^6^^,^^7^ CAZ-AVI combines a third-generation cephalosporin and a non-β-lactam inhibitor, with activity against gram-negative and is currently approved for the treatment of nosocomial pneumonia including VAP, complicated urinary tract, intra-abdominal infections, and bacteraemia associated with any of the above infections.^8^

Antimicrobial resistance poses a threat to public health. Although it occurs naturally, inappropriate use of antibiotics is accelerating the process, resulting in longer average lengths of stays, increased medical costs, and mortality.^9^ The scientific community and public health institutions across the world are calling for action and coordinating strategies to contain the development of resistance.^10^ The World Health Organization (WHO) has proposed several intersectoral measures to prevent and control antimicrobial resistance at different levels.^11^ The Brazilian Ministry of Health endorsed this initiative, creating the PAN-BR, the Brazilian National Plan for Combating Antibiotic-Resistant Bacteria.^12^

The phase III REPROVE study demonstrated the noninferiority of CAZ-AVI to meropenem in the treatment of hospitalized adults with VAP due to Gram-negative pathogens.^4^ Observational studies have shown that CAZ-AVI is safer than polymyxin B and significantly reduces 28-day mortality in patients with carbapenem-resistant VAP.^13^ CAZ-AVI is currently recommended and prescribed by infectious diseases specialists for the treatment of carbapenem-resistant VAP.^3^^,^^4^^,^^7^

To raise awareness among the medical community and promote an evidence-based use of antimicrobials among patients with VAP, a cost-effectiveness analysis was conducted to compare CAZ-AVI with polymyxin B for the treatment of Carbapenem-Resistant *Enterobacterales* (CRE) or Carbapenem-Resistant *Pseudomonas aeruginosa* (CRPa) VAP from the Brazilian National Supplementary Health Agency perspective.^12^

## Material and methods

This cost-effectiveness analysis was reported according to Consolidated Health Economic Evaluation Reporting Standards 2022 (CHEERS 2022).^14^

We conducted a cost-effectiveness analysis using the TreeAge Pro, LLC software (Williamstown, Massachusetts, the United States). A decision tree followed by a Markov model was developed to compare the costs and effectiveness of ceftazidime-avibactam vs. polymyxin B in adult ICU patients with ventilator-associated bacterial pneumonia receiving antibiotics, from the Brazilian National Supplementary Health Agency (Agência Nacional de Saúde Suplementar, ANS) perspective.

The target population for this analysis were adult ICU patients aged 65 years who were diagnosed with CRE or CRPa VAP. Treatment strategies considered in this model were CAZ-AVI, and polymyxin B. Dosages were obtained from the most recent prescribing information available on the Brazilian Health Regulatory Agency (ANVISA) website; for polymyxin B dosage, an average weight of 65 kg was considered as reported by Fang et al., 2021.^8^^,^^13^

### *Model overview*

**Fig. 1:**
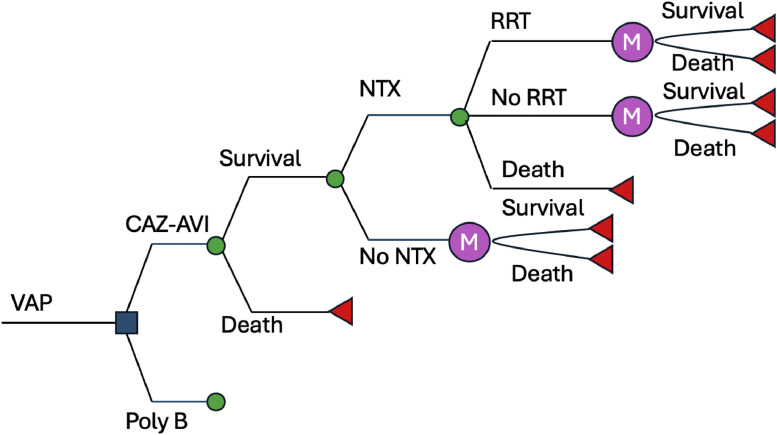
Cost-effectiveness model.

Patients with VAP received either CAZ-AVI or polymyxin B while in the ICU. Following the treatment, nephrotoxicity due to the treatment could have developed or not. Patients who did not need Renal Replacement Therapy (RRT) were discharged home where they could survive or die. For those patients presenting with nephrotoxicity, RRT might have been necessary short- or long-term, then they would receive the therapy and continue in the model as the group without RRT. The time horizon considered was 5 years with 1 year cycles to account for possible antibiotic-related complications (Fig. 1).

### *Model inputs*

#### Costs

Following the ANS perspective, drug prices were obtained from the current version of the Brazilian Drug Market Regulation Chamber (CMED) table.^15^ Factory price +18 % Brazilian tax on circulation of goods and services was used.

Other costs per day and procedure costs were obtained from the 2022 version of the Brazilian Hierarchical Classification of Medical Procedures (CBHPM) table, published literature and healthcare resource utilization costs from a private tertiary hospital in Southeast Brazil (Table 1).^16^

**Table 1:** Model parameters.

Input	Value (base case)	Range (sensitivity analysis)	Distribution	Source
Clinical				
In-hospital 28-day mortality (%)				
CAZ-AVI	8.1	‒	‒	Fang et al., 202,1^13^
Odds ratio increase with Polymyxin B	4.74	1.32 ‒ 16.99	Lognormal	Fang et al., 202,1^13^
Length of stay (days)	17.5	6.92 ‒ 28.08	Normal	Alexander et al., 201,7^27^
NTX (a)	+2.9	‒	‒	Simon et al., 201,9^22^
NTX (%)				
CAZ-AVI	8	4.0 ‒ 20.0	Uniform	Shields et al., 2017,^28^ Van Duin et al., 201,8^29^
Polymyxin B	38	2.8 ‒ 64.8	Uniform	Vardakas et al., 201,7^30^
Treatment Duration (days)	10	7 ‒ 14	Uniform	Drug labels^8^^,^^31^
RRT (%)	7.6	3.4 ‒ 15.9	Beta	Dubrovskaya et al., 2015,^32^ Kubin et al., 201,2^33^
RRT mortality (%)	19.5	14.5 ‒ 24.5	Uniform	Neves et al., 201,9^34^
All-cause mortality (%) (b)	1.24	0.986 ‒ 1.705	Uniform	IBGE 202,1^35^
**Utilities**				
Hospitalization	0.73	0.40‒0.95	Uniform	Tsevat et al., 199,5^36^
Chronic Dialysis	0.59	0.40‒0.80	Uniform	Liem et al., 200,8^37^
Death	0	‒	‒	Briggs et al., 200,6^38^
**Costs (BRL)**				
Drugs (per day)				
CAZ-AVI	2348.57	1761.43 ‒ 2348.57	Uniform	CMED (d)^15^
Polymyxin B (c)	926,4	694.80 ‒ 926.40	Uniform	CMED (d)^15^
Arteriovenous fistula for dialysis	4349.84	3262.40 ‒ 5437.30	Uniform	Base case: CBHPM 2020
				Sensitivity analysis: assumption (e)^16^
Chronic Dialysis (per session)	1011.08	758.31 ‒ 1263.85	Uniform	Base case: CBHPM 2020
				Sensitivity analysis: assumption (e)^16^
ICU cost per day				
No RRT	2836.00	2566 ‒ 8792	Uniform	Base case: Silva Carlos et al., 202,0^39^
				Sensitivity analysis: internal data from a private tertiary hospital
RRT	3662.14	2859.46 ‒ 11,589.67	Uniform	Base case: Silva Carlos et al., 2020,^39^ Dal Secco et al., 2007
				Sensitivity analysis: internal data from a private tertiary hospital
Follow-up visit	224.90	168.67 ‒ 281.12	Uniform	Base case: CBHPM 2020
				Sensitivity analysis: assumption (e)^16^
Monitoring (f)	39.81	29.86 ‒ 49.76	Uniform	Base case: CBHPM 2020
				Sensitivity analysis: assumption (e)^16^
Model				
Time horizon	5 years	1‒10 years	Uniform	Assumption
Discount rate	5 % per year	4 % ‒ 6 %	Uniform	Base case: Brazilian Ministry of Health (g)
				Sensitivity analysis: assumption

BRL, Brazilian Reais; CAZ-AVI, Ceftazidime-Avibactam; CBHPM, Brazilian Hierarchical Classification of Medical Procedures; CMED, Brazilian Drug Market Regulation Chamber; ICU, Intensive Care Unit; NTX, Nephrotoxicity; RRT, Renal Replacement Therapy.

Explanations: (a) For patients with nephrotoxicity requiring RRT, 2.9-days were added to the average length of stay, as reported in Simon et al., 2019; (b) Mortality rate among Brazilians 60‒65 years old, range of values that include the mean age of the participants included in the studies (63 years old); (c) Costs were estimated based on the recommended dosage of 25.000‒30.000 IU/kg/day every 12-hours (polymyxin B package insert) and an average body weight of 65 kg, as reported by Fang et al., 2021; (d) In the sensitivity analysis, the base-case value was varied by −25 %; (e) In the sensitivity analysis, the base-case value was varied by ±25 %; (f) Monitoring included Blood Urea Nitrogen (BUN), Creatinine, and Creatinine Clearance tests (CrCl) on days 3, 7, and 10 during antibiotic therapy, as reported by Silva et al., 2020; (g) Brazilian Ministry of Health Methodological Guidelines for Economic Evaluations.

#### Clinical variables and utilities

In the model, nephrotoxicity due to antibiotic use, proportion of patients in RRT, length of stay, 28-day in-hospital mortality, all-cause mortality, and mortality due to RRT complications were considered. Hospitalization and long-term RRT utilities were included in the model. These parameters were derived from the published literature. Whenever available, Brazilian data was prioritized.

The parameters used in the model, as well as variations for sensitivity analysis, distributions, and sources are available in Table 1.

#### Discount rate

Following the Brazilian Ministry of Health Methodological Guidelines for Economic Evaluations,^17^ a discount rate of 5 % was applied for both costs and effectiveness measures in the base-case scenario.

#### Willingness-To-Pay (WTP) threshold

Following the Brazilian Ministry of Health recommendations about the adoption of a WTP threshold for the Brazilian Unified Health System, a WTP of BRL 40,000.00 / Quality-Adjusted Life Years (QALY) gained was considered in the base-case analysis. However, considering that VAP followed or not by nephrotoxicity are severe conditions, a scenario analysis was conducted using a WTP threshold of BRL 120,000.00/QALY gained. We considered the disease severity according to recommendations from the national commission for incorporating technologies into the SUS (CONITEC), since there is no formal WTP threshold defined by Supplementary Healthcare Agency (ANS).^18^

#### Sensitivity analysis

A one-way Deterministic Sensitivity Analysis (DSA) was performed to assess the model's uncertainties and to identify which variables most impacted the results obtained in terms of incremental costs. In the sensitivity analysis, the varied parameters included cost of drugs, duration of drug regimens, clinical parameters (nephrotoxicity, 28-day in-hospital mortality, all-cause mortality, and RRT), discount rates, and time horizon. Each parameter range is available on Table 1. To test the robustness of the model, a Probabilistic Sensitivity Analysis (PSA) was performed. In the Monte Carlo simulation, multiple parameters were varied simultaneously and randomly in 1000 iterations.

#### Assumptions

In the model, it was assumed that : i) Standard of care during hospitalization was provided for all patients equally; ii) Concomitant use of other antibiotics was expected in both treatment arms but was not considered in the model given the variety of regimens and pathogens; iii) Patients have not switched between the compared treatments during the hospitalization; iv) All patients had started the model with creatinine clearance levels > 50 mL/min/1.73 m^2^ and did not need dose adjustments.

## Results

In the base-case analysis, treatment with CAZ-AVI was more expensive than polymyxin B but resulted in more QALYs gained. At the WTP threshold of BRL 40,000.00/QALY gained, CAZ-AVI was cost-effective compared to polymyxin B, with an Incremental Cost-Effectiveness Ratio (ICER) of BRL 35,298.65 per QALY gained (Table 2).

**Table 2:** Base-case analysis results.

Strategy	Cost	Incremental cost	Effectiveness	Incremental Effectiveness	ICER
Polymyxin B	115,035	10,376	0.31	0.29	35,298.65
CAZ-AVI	125,412		0.61		

ICER, Incremental Cost-Effectiveness Ratio.

**Fig. 2:**
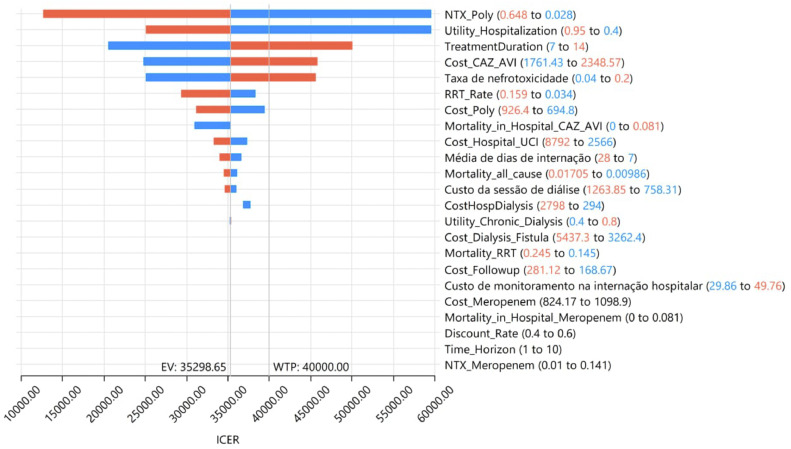
Tornado diagram (WTP = 40,000.00).

**Fig. 3:**
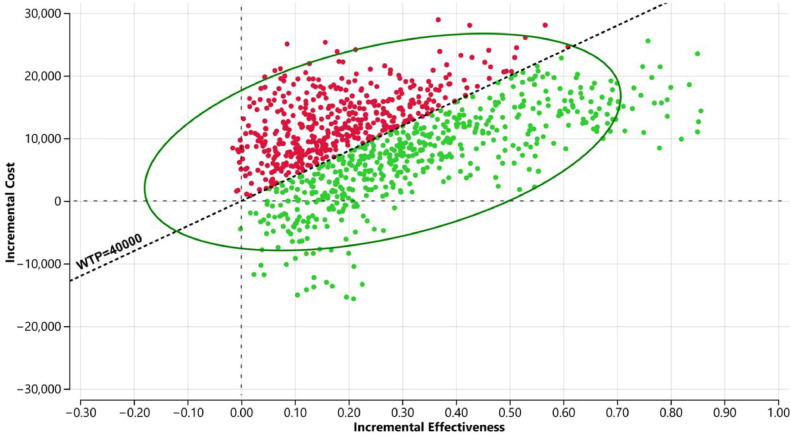
Probabilistic sensitivity analyses scatterplot (WTP = 40,000.00).

In the one-way DSA, the variables that most influenced the models were the proportion of patients in the polymyxin B group experiencing nephrotoxicity, the utility of hospitalization, and treatment duration (Fig. 2). The results of the PSA are shown in Fig. 3. At the WTP threshold of BRL 40,000.00/QALY gained, CAZ-AVI was cost effective in approximately 55 % of the iterations.

**Fig. 4:**
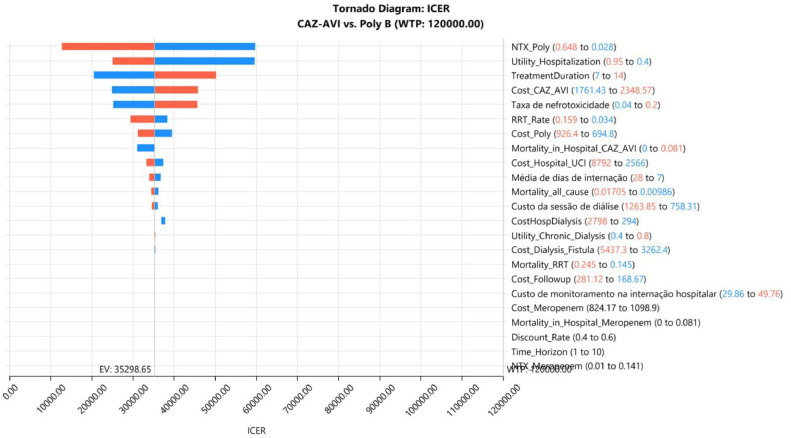
Tornado diagram (WTP = 40,000.00).

**Fig. 5:**
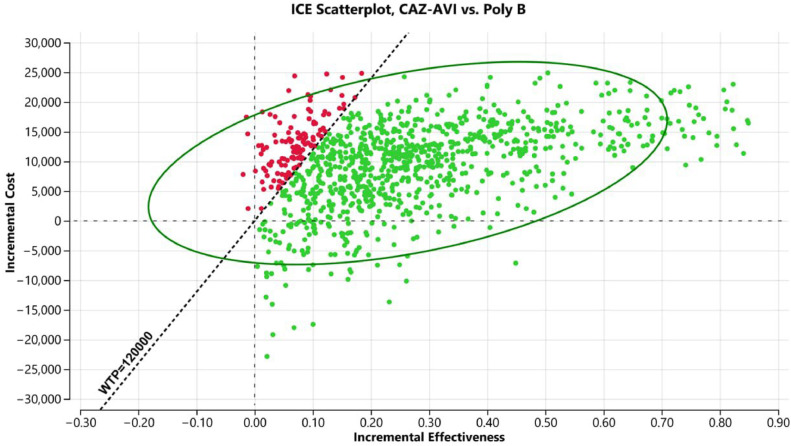
Probabilistic sensitivity analyses scatterplot (WTP = 120,000.00).

Considering that VAP followed by nephrotoxicity and RRT are severe conditions, we also explored a scenario adopting the WTP threshold of BRL 120,000/QALY gained. In this set of analyses, the ICER and one-way DSA results remained unchanged, but in the probabilistic sensitivity analysis, CAZ-AVI was cost-effective in about 89 % of the iterations (Fig. 4, Fig. 5).

## Discussion

Our study attested the cost-effectiveness of CAZ-AVI considering different WTP thresholds. In the most conservative scenario, CAZ-AVI was cost-effective in at least 55 % of the iterations. Furthermore, despite the increase in cost, CAZ-AVI has substantially improved QALYs. An important point depicted in the tornado sensitivity analysis is the clear impact of nephrotoxicity of polymyxin B regimens in the cost-effectiveness.

Currently, the National Supplementary Health Agency (ANS) does not have a cost-effectiveness threshold to support decision-making on the incorporation of new technologies in the Brazilian private health system. In August 2023, a symposium took place on this topic, in which some participants defended the use of the same threshold as the National Commission for the Incorporation of Technologies in the Unified Health System (CONITEC), since this is based on Gross Domestic Product (GDP) of the country as a whole, without stratification by public or private system.^19^ However, other participants argued that the cost-effectiveness threshold value of the ANS should be higher than that of the SUS. As there is still no decision on the value of the cost-effectiveness threshold in the ANS, in the present model, the analyses were carried out using CONITEC’s cost-effectiveness threshold, in which CAZ-AVI proved to be cost-effective both considering the threshold of BRL 40,000/QALY, as well as at the threshold of BRL 120,000/QALY. The latter can also be used since despite not being a rare disease, it is a potentially a fatal clinical condition, for which it is believed that the expenditure of greater volumes of resources is appropriate. However, it is noteworthy that in both cost-effectiveness threshold scenarios and in all sensitivity analyses, CAZ-AVI was cost-effective, revealing the robustness of the model.

To the best of our knowledge, few cost-effectiveness models evaluated CAZ-AVI for the treatment of VAP^20^^,^^21^ and only one model compared CAZ-AVI vs. polymyxins for the treatment of carbapenem-resistant VAP.^21^, ^22^, ^23^ Simon et al., 2019 developed a cost-effectiveness model that compared CAZ-AVI vs. colistin regimens over a 5-year time horizon from a U.S. health care system perspective.^22^ They also found that CAZ-AVI was cost-effective compared to colistin considering a WTP threshold commonly accepted in the U.S. (USD 100,000 and USD 150,000/QALY gained), with an ICER of USD 95,000/QALY gained.^22^ The model structure was similar to ours, but it also included costs and utilities of long-term care, which was not feasible in our model due to the idiosyncrasies of the Brazilian National Supplementary Health Agency. Despite these differences, our results are aligned with international data, and it supports our finding that CAZ-AVI is cost-effective compared with a polymyxin B, especially when the latter is associated with higher rates of nephrotoxicity and need for RRT.^13^

Our analyses have several important limitations that should be considered when interpreting results. First, as with any modeling study, our model inputs are constrained by the quality of existing data. Specifically, CAZ-AVI’s effectiveness is based on one randomized clinical trial and small number of observational studies. Long-term health and economic outcomes were based on literature that is not specific to carbapenem-resistant infections. To account for this uncertainty, we conducted extensive sensitivity analyses and varied model inputs across a wide range of plausible values. It is worth noting that the model considered patients with carbapenem-resistant infections, therefore, meropenem would not be a viable option. Furthermore, polymyxins have been frequently used as the first option among well-established alternatives of care for the treatment of carbapenem-resistant infections. Other options, such as meropenem-vaborbactam and cefiderocol are not currently registered by ANVISA, the Brazilian regulatory agency. Imipenem-relebactam was not available in the Brazilian market by the time the model was developed, thus it was not considered in the economic evaluation. Therefore, we consider that the comparison between CAZ-AVI vs. Polymyxin B is fairly representative of the therapeutic landscape in Brazil. It is possible that cost-effectiveness results would be less favorable for carbapenem-sensitive infections. Budget impact analysis was out of the scope of this study from the ANS perspective, but it is important that future studies evaluate it. Our model also has some strengths. The model was validated by an infectious disease specialist, and it truly represents the pragmatic course of the disease. Variables related to both costs and effectiveness are aligned with previously published cost-effectiveness analyses in other contexts/countries. Moreover, considering that economic analysis generally presents a low external validity (i.e., it is difficult to adapt a result from external contexts to ours), our model is the first study considering a Brazilian scenario.

The excessive use of new antimicrobials exerts selective pressure, negatively impacting bacterial resistance. The treatment of infections caused by resistant isolates is associated with higher costs and increased resource utilization, as well as a higher risk of unfavorable clinical outcomes.^24^ In this context, national data from ANVISA on antimicrobial resistance from 2023 indicate a 28.4 % resistance rate to CAZ-AVI in bacterial isolates from primary bloodstream infections.^25^ Therefore, despite the cost-effectiveness of CAZ-AVI, the introduction of new antimicrobials into clinical practice requires caution and should preferably occur within the framework of an antimicrobial stewardship strategy to preserve treatment efficacy.^26^

## Conclusion

The cost-effectiveness model has shown that CAZ-AVI was cost-effective when compared with Polymyxin B, regardless of the WTP threshold adopted (BRL 40,000 or 120,000/QALY gained). Despite being more costly, CAZ-AVI was superior in terms of effectiveness, resulting in ICER = BRL 35,298.65/QALY gained. The parameters that most influenced the model were RRT rate in patients receiving Polymyxin B, utility of hospitalization and treatment duration. In the PSA, CAZ-AVI was cost-effective in 55 % and 89 % of the interactions, adopting WTP thresholds of BRL 40,000 and 120,000/QALY gained, respectively. Thus, CAZ-AVI seems to be the strategy that would result in the most favorable allocational efficiency in the context of these analyses.

## Funding

JM, FP, HAO Jr are employees of the Hospital Alemão Oswaldo Cruz, which received funding from Pfizer in connection with the development of this manuscript. DVP is shareholder and employee of Pfizer. LCCF is employee of Pfizer.

This study was sponsored by Pfizer Brasil.

Conceptualization and review of the manuscript. The funding source had no role in the design and conduct of the study, access and collection of data, and analysis and interpretation of data.

## Conflicts of interest

The authors declare no conflicts of interest.
